# Wnt/β-catenin Signaling Pathway Regulates Specific lncRNAs That Impact Dermal Fibroblasts and Skin Fibrosis

**DOI:** 10.3389/fgene.2017.00183

**Published:** 2017-11-21

**Authors:** Nathaniel K. Mullin, Nikhil V. Mallipeddi, Emily Hamburg-Shields, Beatriz Ibarra, Ahmad M. Khalil, Radhika P. Atit

**Affiliations:** ^1^Department of Biology, Case Western Reserve University, Cleveland, OH, United States; ^2^Department of Genetics and Genome Sciences, School of Medicine, Case Western Reserve University, Cleveland, OH, United States; ^3^Department of Dermatology, Case Western Reserve University, Cleveland, OH, United States

**Keywords:** gene expression, *Mmp10*, *Wincr1*, *Wincr2*, dermis, development

## Abstract

Wnt/β-catenin signaling is required for embryonic dermal fibroblast cell fate, and dysregulation of this pathway is sufficient to promote fibrosis in adult tissue. The downstream modulators of Wnt/β-catenin signaling required for controlling cell fate and dermal fibrosis remain poorly understood. The discovery of regulatory long non-coding RNAs (lncRNAs) and their pivotal roles as key modulators of gene expression downstream of signaling cascades in various contexts prompted us to investigate their roles in Wnt/β-catenin signaling. Here, we have identified lncRNAs and protein-coding RNAs that are induced by β-catenin activity in mouse dermal fibroblasts using next generation RNA-sequencing. The differentially expressed protein-coding mRNAs are enriched for extracellular matrix proteins, glycoproteins, and cell adhesion, and many are also dysregulated in human fibrotic tissues. We identified 111 lncRNAs that are differentially expressed in response to activation of Wnt/β-catenin signaling. To further characterize the role of mouse lncRNAs in this pathway, we validated two novel Wnt signaling- Induced Non-Coding RNA (Wincr) transcripts referred to as *Wincr1* and *Wincr2*. These two lncRNAs are highly expressed in mouse embryonic skin and perinatal dermal fibroblasts. Furthermore, we found that *Wincr1* expression levels in perinatal dermal fibroblasts affects the expression of key markers of fibrosis (e.g., *Col1a1* and *Mmp10*), enhances collagen contraction, and attenuates collective cell migration. Our results show that β-catenin signaling-responsive lncRNAs may modulate dermal fibroblast behavior and collagen accumulation in dermal fibrosis, providing new mechanistic insights and nodes for therapeutic intervention.

## Introduction

Wnt/β-catenin signaling has a diverse role in both embryonic mouse skin development and in human skin diseases such as pilomatricomas, dermal hypoplasia, and fibrosis (Wang et al., 2007; Lam and Gottardi, 2011; Hamburg and Atit, 2012; Lim and Nusse, 2012). β-catenin is a key transducer of the Wnt/β-catenin signaling pathway and a regulator of transcription (van Amerongen and Nusse, 2009; Schuijers et al., 2014; McCrea and Gottardi, 2016). Cell type and context-specific target gene expression provide specificity for the diverse functions of the Wnt signaling pathway (Nakamura et al., 2009; Nusse and Clevers, 2017). Elucidating the downstream modulators of the Wnt signaling pathway is critical to our understanding of how this pathway influences distinct cell types in development and disease.

Dermal fibroblasts are key contributors to hair follicle development, regional identity, skin patterning, wound healing, and skin fibrosis (Chang et al., 2002; Eames and Schneider, 2005; Rendl et al., 2005; Enzo et al., 2015). We have previously demonstrated that dermal Wnt/β-catenin activity is required for mouse embryonic dermal fibroblast identity and hair follicle initiation (Atit et al., 2006; Ohtola et al., 2008; Tran et al., 2010; Chen et al., 2012). Activation of the Wnt signaling pathway in humans is also a common feature in fibrosis of varying organs such as lung, liver, kidney, and skin (Enzo et al., 2015). We found that sustained Wnt/β-catenin activation in dermal fibroblasts is sufficient to cause dermal fibrosis in the adult mouse (Akhmetshina et al., 2012; Hamburg and Atit, 2012; Mastrogiannaki et al., 2016). Transcriptome analysis of mouse fibrotic dermis showed an increase in mRNA levels of regulatory genes, such as *Col7a1, Ccn3/Nov, Biglycan*, and *Matrix Metalloproteinase 16 (Mmp16)*, a subset of which are also up-regulated in human skin fibrosis and tumor stroma (Hamburg-Shields et al., 2015). Thus, studying the various mechanisms of β-catenin-mediated gene regulation will provide new insights into how context-specific transcriptional targets are activated and repressed in skin development and disease.

In recent years, lncRNAs have emerged as crucial intermediate facilitators for a variety of cellular pathways and gene expression networks (Rinn and Chang, 2012; Wan and Wang, 2014). Regulatory lncRNAs are defined as >200 nt-long RNA molecules readily present within both the cytosol and nucleus but lacking protein-coding capacity. Various studies have implicated lncRNAs in directly facilitating gene expression, both in *cis* and in *trans* (Khalil et al., 2009; Moran et al., 2012). Furthermore, studies have documented the existence of differentially expressed lncRNAs within specific fibrotic conditions (Huang et al., 2015; Micheletti et al., 2017; Piccoli et al., 2017; Qu et al., 2017). Recently, TGFβ-responsive lncRNAs have been shown to directly control the expression of fibrotic genes, demonstrating a role for lncRNAs in a pathway with a similar function as Wnt/β-catenin signaling (Fu et al., 2016; Wang et al., 2016). An increasing number of studies are investigating the direct role that lncRNAs play in influencing Wnt/β-catenin signaling (Fan et al., 2014; Vassallo et al., 2015; Wang et al., 2015; Ma et al., 2016).

Wnt/β-catenin signaling-induced lncRNAs that function as downstream effectors of Wnt signaling to modulate gene expression have yet to be fully characterized. Here, we expressed a stabilized version of β-catenin protein from the endogenous locus in neonatal mouse primary dermal fibroblasts to identify Wnt/β-catenin signaling-responsive mRNAs and lncRNAs. We functionally characterized one of the most differentially expressed lncRNAs, *GM12603*, referred to as *Wincr1* in the context of fibrotic gene expression and fibroblast behavior. Our results show that *Wincr1* has gene-regulatory and functional roles in key behaviors of dermal fibroblasts such as collective cell migration and collagen contraction.

## Materials and Methods

### Animals and Ethics

*Ctnnb1*^Δ^*^ex3^*^/+^ (Harada et al., 1999), *Engrailed1Cre (En1Cre)* (Kimmel et al., 2000), *Gt(ROSA)26Sor^tm1(rtTA,EGFP)Nagy^ (R26rtTA)* Jax labs stock: 005670 (Belteki et al., 2005), *Teto-deltaN89* β*-catenin* (Mukherjee et al., 2010) were maintained on mixed genetic background (CD1, C57Bl6) and genotyped as previously described. Triple transgenic *Engrailed1Cre/*+; *Rosa26rtTA/+; Teto-deltaN89* β*-catenin/*+ experimental mice were generated. For each experiment, a minimum of three mutants with litter-matched controls were studied except where otherwise noted. Animals of both sexes were randomly assigned to all studies. Case Western Reserve Institutional Animal Care and Use Committee approved all animal procedures in accordance with AVMA guidelines (Protocol 2013-0156, approved 21 November 2014, Animal Welfare Assurance No. A3145-01).

### Collection and Culture of Primary Dermal Fibroblasts

Whole ventral skin from *Ctnnb1*^Δex3/+^ mice was dissected from the trunk of postnatal days 4–7 (P4-7) mice and minced (Harada et al., 1999). Skin was incubated in Dulbecco’s Modified Eagle’s Medium: Nutrient Mixture F-12 (DMEM/F12) (Thermo Fisher Cat. No. 11320) and 50 mg/mL Liberase DL (Roche Cat. No. 5401160001) at 37°C under constant rotation for 1 h. Dermis was dissociated by vigorous pipetting and passing through an 18 gauge needle (BD Cat. No. 305196). Cells from each animal were cultured individually in complete growth media (DMEM (Thermo Fisher Cat. No. 11995065) + 10% Fetal Bovine Serum (FBS) (Thermo Fisher Cat. No. 10082147) + 1% Penicillin/Streptomycin (Invitrogen Cat. No. 15140122) + 1% Antibiotic-Antimycotic) (Invitrogen Cat. No. 14240062) in 5% CO_2_ except where otherwise noted. After 90 min, media was removed and replaced with fresh culture media. After two passages, Adenovirus was administered in basal DMEM (no FBS). Dermal fibroblasts were infected with Adenovirus-Cre (366-500 MOI) or Adenovirus-GFP (366 MOI) (Adenovirus purchased from University of Iowa). After 48 h, recombination of *Ctnnb1* Exon 3 was confirmed with site specific PCR under standard conditions. Primers *Neo PMR* (5′ AGACTGCCTTGGGAAAAGCG 3′) and *Cat-AS5* (5′ ACGTGTGGCAAGTTCCGCGTCATCC 3′) were used to identify the targeted allele with a ∼500 bp amplicon and *GF2* (5′GGTAGGTGAAGCTCAGCGCAGAGC 3′) and *Cat-AS5* identified the recombined allele with an expected amplicon of ∼700 bp (Harada et al., 1999).

For *in vivo* induction of stabilized β-catenin expression in *Engrailed1Cre/*+; *Rosa26rtTA/*+; *Teto-deltaN89*β*-catenin/*+ mice, pregnant dams were given 80 μg of doxycycline (Sigma Cat. No. D9891) per gram of body weight by intraperitoneal injection at embryonic day 12.5 (E12.5) and embryonic cranial and dorsal dermal skin were harvested at E13.5. *In vitro* stabilized β-catenin expression was induced by treating *Engrailed1Cre/*+; *Rosa26rtTA/*+; *Teto-deltaN89*β*-catenin/*+ P4 ventral dermal fibroblasts cells with 2 μg/mL doxycycline in complete growth media for 4 days. For induction-reversal experiments, duplicate cultures were then switched to complete growth media without doxycycline for 48 h prior to RNA isolation.

### Whole-Genome RNA Sequencing

Total RNA was extracted from embryonic tissues *in vivo* and dermal fibroblasts *in vitro* using TRIzol reagent (Thermo Fisher Cat. No. 15596026). RNA was isolated using the RNeasy MinElute kit (Qiagen Cat. No. 74204) with DNAse1 (Qiagen Cat. No. 79254) treatment, following the manufacturer’s protocol. RNA concentration and quality were measured using the NanoDrop 8000 UV-Vis Spectrophotometer.

Libraries were prepared by the CWRU Genomics Sequencing Core, using the True-Seq Stranded Kit (Illumina). Paired-end sequencing was carried out on the Illumina HiSeq 2500 platform. Resulting 100 bp reads were mapped to the mm10 mouse genome release using TopHat and Cufflinks. Mapped raw reads were counted, normalized to total mapped reads, and used for differential gene expression (Cuffdiff and Seqmonk standard settings). Differential expression was defined as an absolute fold change greater than 2, and Benjamini–Hochberg adjusted *P*-value less than 0.05. Normalized mapped reads are available on Gene Expression Omnibus (GSE103870) using the private token from the editor.

### Quantitative PCR and Primers

Total RNA was extracted from P4 ventral dermal fibroblasts between passage 3–6 as mentioned above. cDNA was generated using the Invitrogen High Capacity RNA-to-cDNA Reverse Transcription Kit (Thermo Fisher Cat. No. 4374966). Relative mRNA quantities of select genes were determined using the Applied Biosystems StepOnePlus Real-Time PCR System (Life Technologies Cat. No. 4376600) and the Δ*C*t or ΔΔ*C*t method where applicable (Livak and Schmittgen, 2001; Schmittgen and Livak, 2008). In all plots, sample and control RQs were normalized to the mean RQ of the control group. *Axin2* quantity was measured relative to the reference gene *ActB* using Taqman probes from Thermo Fisher (Mm00443610_m1 and Mm02619580_g1, respectively) or *HPRT* (mm1545399_m1). *Wincr1* isoform1 (F: TGATCCCACTGAAAATGCTG, R:GGTGATTTGACCTGCCATCT) and *Wincr2* (F:GGCCTGGATAGAGGTCTCC, R:TAGTTCTCTCCATCGGTTTCC) quantities were measured relative to reference gene *RPL32* (F:TTAAGCGAAACTGGCGGAAAC, R:TTGTTGCTCCCATAACCGATG) using custom-designed primers (Invitrogen) and SyBr Green reagents(Invitrogen Cat. No. 4367659). *Mmp10 and Col1a1* quantities (Mm01168399_m1 and Mm00801666_g1, respectively) were measured relative to reference gene *ActB* expression, using TaqMan Gene Expression Master Mix (Thermo Fisher Cat. No. 4369016). Non-coding gene primers were designed using Primer3Plus^1^. Primer sequences for *RPL32* and *Mmp10* were acquired through the MGH Primer Bank Site^2^. Primer sequences for *Col1a1* were identified previously (He et al., 2005).

Statistics were performed using GraphPad Prism 7. For experiments in which cells from the same animal were used in control and experimental conditions, a paired *t*-test was used. In all other instances, an unpaired *t*-test was used. Significance for all purposes was defined as ^∗^*P*-value ≤ 0.05, ^∗∗^*P*-value ≤ 0.01, ^∗∗∗^*P*-value ≤ 0.001, ^∗∗∗∗^*P*-value ≤ 0.0001. Paired samples are shown as dots connected by a line where applicable. Expression across different tissues are shown as mRNA levels relative to reference gene (2^-ΔCt^). In all other plots, individual relative quantities (2^-ΔΔCt^) are shown along with mean and standard error of the mean.

### Bioinformatic Analysis

Heatmaps were generated using all mRNA or lncRNA genes considered significantly differentially expressed [abs(fold change) > 2, adjusted *P*-value < 0.05] between GOF and control samples. Color was assigned to each sample on a basis of *Z*-score of fragments per kilobase mapped (FPKM) compared to other samples’ FPKM of the same gene (row *Z*-score). Heatmaps were clustered hierarchically based on the aggregate differential gene expression profiles using the DESeq2 package in R.

DAVID Functional Annotation Clustering^3^ was performed on all differentially expressed protein-coding genes [abs(fold change) > 2, adjusted *P*-value < 0.05]. Gene lists were entered as Gene Symbols. All settings were default. The top five ranked clusters are shown, with clusters enriched among up-regulated genes shown as positive and clusters enriched among down-regulated genes shown as negative.

Overrepresentation of predicted transcription factor binding sites was determined using oPOSSUM 3.0 Single Site Analysis (SSA)^4^ (Ho Sui et al., 2007; Kwon et al., 2012). Gene lists were loaded into the browser-based software by gene symbol, and putative transcription factor binding sites (TFBS) were scored based on their overrepresentation in the areas surrounding the transcription start site (TSS) of such genes, as compared to prevalence in the rest of the genome. All 29,347 genes in the oPOSSUM database were used as background. For analysis of TFBS enrichment around lncRNAs, custom FASTA files were generated for the promoter sequence of each gene. Proximity to TSS was defined as ±5 kb for all analysis. All JASPAR PBM profiles were queried. Each TFBS was assigned a *Z*-score and a Fisher score for overrepresentation. TFBS were then plotted according to these scores using GraphPad Prism 7. Thresholds were determined as follows: *Z*-score *- Mean* + *(2 × Standard Deviation)*, Fisher score -*Mean* + *Standard Deviation* as previously described (Kwon et al., 2012).

Gene Set Enrichment Analysis (GSEA) was used to identify known gene expression signatures similar to the differential expression of genes in GOF dermal fibroblasts (Mootha et al., 2003; Subramanian et al., 2005). GSEA was run as a Java Applet. MSigDB C2 Curated Gene Sets were queried. Gene sets smaller than 15 genes and larger than 500 genes were excluded from the analysis and 1000 *gene_set* permutations were used. All other settings remained default.

The human matrisome gene list was accessed through the MIT Matrisome Project at MatrisomeDB^5^. The intersection of this list with others was performed in Microsoft Excel.

Coexpression networks were constructed using Cytoscape version 3.4.0 (Shannon et al., 2003). Candidate genes were selected on the basis of significant differential upregulation (adjusted *P*-value < 0.05 and fold change > 2) and inclusion in the Matrisome gene list (see above). An FPKM table was constructed in Microsoft Excel consisting of these candidate genes as well as *Axin2, Wincr1*, and *Wincr2*. This table was loaded into Cytoscape as an unassigned table. The plugin “Expression Correlation” was used (with a Low Cutoff of -1 and a High Cutoff of 0.6) to generate a correlation network. The built-in tool “Network Analyzer” was used to further analyze the network for node degree. A custom style was created to visualize the network, in which edge color correlates with strength of correlation (red:high CC). Node size correlated with number of first neighbors (degree).

### Human Fibrotic Disease Study Expression Analysis

Microarray data from nine human studies of various fibrotic conditions were analyzed using Gene Expression Omnibus (GEO). Data were analyzed using the GEO2R tool. Genes with average fold change > 1.5 and adjusted *P*-value < 0.05 were considered significantly differentially expressed.

### *In Vitro* Lentivirus Overexpression and Gapmer Knockdown of *Wincr1*

Full-length isoform 1 of *Wincr1* was assembled from synthetic oligonucleotides and PCR products and subcloned into pMA-T (Life Technologies, Ref. No. 1725930). *Wincr1* lentivirus was generated by subcloning *Wincr1* from pMA-T into the Nco1-EcoRV region of pENTR4 (Thermo Fisher Cat. No. A10465) vector and then swapping it into the pGK destination vector (Addgene Cat. No. 19068). Lentivirus was generated in 293T (f-variant) cells (ATCC Cat. No. 3216) by simultaneously transfecting pGK Destination Vector containing *Wincr1* (4.5 μg), PMD2G helper plasmid (1.6 μg) (Addgene Cat. No. 12259), and pCMV-dR874 plasmid (3.2 μg), using Lipofectamine 3000 reagent (18 μL) (Invitrogen Cat. No. L30000015) in 1.5 mL Opti-MEM (Thermo Fisher Cat. No. 31985062) media in a 6 cm dish. Cells were selected in Puromycin (2 μg/mL) (Sigma Cat. No. P8833). Virus was collected from 293T conditioned cell media at 24 and 52 h after transfection, clarified with 45 μm pore filter (PES membrane, Thermo Fisher Cat. No. 7252545) and frozen. Lentivirus conditioned media was titered on 293T cells at 3.25–50% dilutions. After 3 days, infected cells were selected by treatment with Puromycin (2 μg/mL) for 3 days. Stable expressing P4 mouse ventral dermal fibroblast cells lines were propagated for further experiments. *Wincr1* overexpression was confirmed by qRT-PCR as described.

*Wincr1* knockdown was achieved with custom LNA-GapmeRs designed and synthesized by Exiqon^6^. Sequences targeting *Wincr1* (GACTAGGATGATAGAT) and a negative (scrambled) control (AACACGTCTATACGC) were acquired. LNA GapmeRs were reconstituted to 50 μM in tissue culture-grade water as per manufacturer’s instruction. Transfection was carried out using Lipofectamine 3000 Reagent in Opti-MEM. Dermal fibroblasts were seeded in a 12-well cell culture treated plate and transfected in 500 μL of Opti-MEM containing 2 μL Lipofectamine 3000 and a final LNA GapmeR concentration of 50 nM. Active transfection was carried out for 6 h, at which time the Lipofectamine 3000 containing media was replaced with complete growth media. Additional LNA GapmeRs were added for a final concentration of 100 nM. After 48 h of unassisted transfection, cells were harvested and RNA was extracted and analyzed for gene expression changes as described above. The knockdown of *Wincr1* expression was confirmed by qRT-PCR.

### Proliferation, Migration, and Contraction Functional Assays

To measure cell proliferation, a standard growth curve assay was performed on P4 ventral dermal fibroblasts between passage 4–6 as described above. 30,000 cells were plated in duplicate into a 12 well plate, with cell number being assessed using Trypan blue (Invitrogen/Gibco Cat. No. 1520061) exclusion on the Cell Countess (Invitrogen Cat. No. C10281). Collective cell migration was assessed using a qualitative scratch assay (Liang et al., 2007). Briefly, a 200 μL pipet tip was used to scratch the monolayer and create a 400 μm gap. Images of cells were taken at time (T) 0, 15, and 22 h. Images were taken on Leica S6D microscope with MC120 HD camera with Leica software. Cell contraction was assessed using the Cell Contraction Assay Kit (Cell Biolabs Inc., CBA-201), following manufacturer instruction. Images of cells were taken with Olympus IX71 microscope with Olympus BX60 camera using Olympus DP controller software. All images were analyzed in Image J software (Schneider et al., 2012).

## Results

### Global RNA Expression Is Altered after β-catenin Stabilization in Dermal Fibroblasts

**FIGURE 1:**
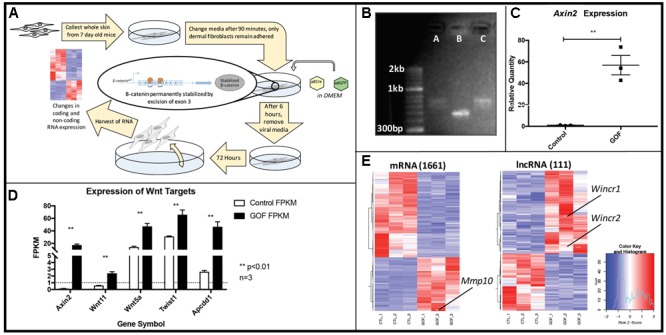
*In vitro* genetic recombination of β*-catenin* (*Ctnnb1*^Δ^*^ex3^)* in dermal fibroblasts results in a strong Wnt signaling expression signature as well as global expression changes in coding and non-coding genes. **(A)** Schematic of the workflow **(B)** *Ctnnb1*^Δ^*^ex3^* recombination by Adenovirus-Cre (AdCre) infection was confirmed by *Ctnnb1* exon3 site-specific PCR in lanes A and C, compared to the control lane B, infected with Adenovirus-GFP. **(C)** Relative mRNA quantity of *Axin2*, a direct β-catenin target is significantly higher in β-catenin stabilized gain of function dermal fibroblasts (GOF) (*P*-value = 0.0035, *n* = 3). **(D)** Known β-catenin targets are higher in GOF dermal fibroblasts by RNAseq (*n* = 3). **(E)** Heat map showing hierarchical clustering (based on entities and samples) of all differentially expressed mRNAs (1661) and lncRNAs (111) (*P*-value < 0.05, fold change > 2) in response to β-catenin stabilization.

To identify coding and non-coding RNAs downstream of Wnt/β-catenin signaling, we infected neonatal dermal fibroblasts carrying *Ctnnb1*^Δ^*^ex3^*^/+^ with Adenovirus Cre (Ad-Cre) (**Figure 1A**). Recombination of Exon 3 of *Ctnnb1* produces a stabilized form of β-catenin protein, referred to as Gain of Function (GOF), which lacks the phosphorylation site for degradation and constitutively activates the canonical Wnt signaling pathway. Using site-specific primers, we confirm the consistent and effective Ad-Cre-mediated excision of *Ctnnb1* Exon 3 by 48 h post-infection, compared to fibroblasts carrying the same transgene and transduced with Adenovirus GFP (**Figure 1B**) (Harada et al., 1999). *Axin2*, a well-established direct target of β-catenin (Jho et al., 2002), was up-regulated by 40 to 70-fold in cells at 72 h post-infection across all GOF samples (**Figure 1C**) further demonstrating successful excision of *Ctnnb1* Exon 3 and activation of the pathway.

Next, we isolated total RNA and performed expression profiling on control and GOF neonatal dermal fibroblasts (*n* = 3) by whole-genome RNA-sequencing at 72 h post Ad-Cre infection (deposited in GEO, GSE103870). We quantified gene expression using FPKM (see Materials and Methods) and further verified GOF status by examining FPKM values of several well-known Wnt/β-catenin signaling targets in dermal fibroblasts *in vivo*, such as *Wnt5a, Wnt11, Apcdd1*, and *Twist2* (Budnick et al., 2016). All target gene expression levels were significantly higher across all three GOF samples, serving as quality control for the sample preparation and sequencing (**Figure 1D**). Within expressed mRNAs and lncRNAs (FPKM > 1), GOF samples independently clustered together via Pearson correlation against the controls (**Supplementary Figure S1**). In total, we identified 1,661 mRNAs and 111 lncRNAs that were differentially expressed across all GOF samples (fold change > 2, adjusted *P*-value < 0.05) (**Figure 1E**). These findings demonstrate that Wnt signaling differentially regulates lncRNAs as well as mRNAs in dermal fibroblasts.

### Stabilization of β-catenin Leads to Dysregulation of Matrisome Genes Relevant to Human Fibrosis

**FIGURE 2:**
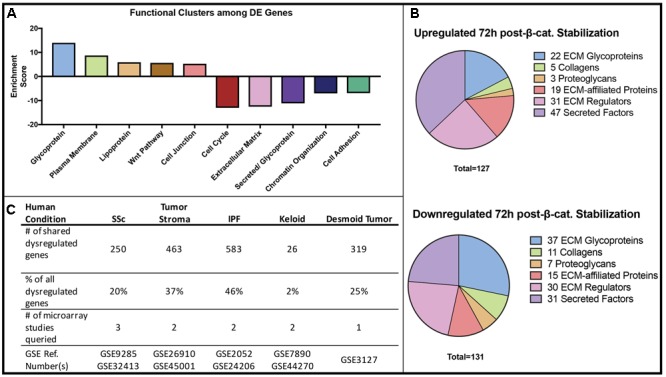
Gene Ontology and disease comparison analysis of all β-catenin responsive genes. **(A)** The functional annotation (DAVID) of differentially expressed (DE) genes in β-catenin stabilized GOF. **(B)** A defined matrisome gene dataset (Naba et al., 2012) overlapped with differentially expressed genes in GOF yielded 127 up-regulated and 131 down-regulated matrisome genes consisting of ECM glycoproteins, secreted factors and ECM-regulators. **(C)** Differentially expressed genes in GOF dataset are also dysregulated in a variety of human fibrotic conditions.

We performed Gene Ontology (GO) Analysis for all differentially expressed mRNAs. These analyses identified key functional groups affected by Wnt/β-catenin signaling in dermal fibroblasts. Through DAVID, the differentially expressed mRNAs were grouped by functional clusters, with positive enrichment scores indicating functional clusters comprised of up-regulated genes and negative scores indicating clusters of down-regulated genes (**Figure 2A**). The highest scoring functional clusters enriched in up-regulated genes included Glycoprotein, Membrane, Cell junction, and Wnt signaling (fold change > 2, adjusted *P*-value < 0.05). Functions including Extracellular Matrix and Secreted/Glycoprotein were also highly enriched among those genes that were down-regulated in GOF dermal fibroblasts (fold change, <0.5-adjusted *P*-value < 0.05).

Since DAVID functional clustering analysis highlighted a strong overrepresentation of ECM genes, we used an existing gene set consisting of matrix encoding and related genes or “matrisome” (Naba et al., 2012), to further annotate the categories of ECM-associated genes. We identified 127 significantly up-regulated and 131 significantly down-regulated matrisome genes in GOF samples compared to controls. Of all significantly dysregulated genes in GOF samples, 9.1% were included in the matrisome gene list. Both up-regulated and down-regulated gene sets revealed expression changes in ECM Glycoproteins, Secreted factors, and ECM-regulators (**Figure 2B**). Of the 258 differentially expressed matrisome genes, 59 were annotated as ECM glycoproteins, 16 are Collagen proteins, 10 are Proteoglycans and 34 are ECM affiliated genes, while the rest serve ECM regulatory functions. Within the various functions of differentially expressed genes, the ECM-encoding and matrisome-related genes are of note due to their function in fibrogenesis.

We utilized Gene Set Enrichment Analysis (GSEA) to identify biological signatures enriched in GOF samples (Subramanian et al., 2005). We input 3114 mouse genes (adjusted *P*-value < 0.05) expressed in our control and GOF fibroblasts, 2371 of which corresponded to human genes with microarray identifiers in the GSEA database (**Supplementary Figure S3A**). The top four gene sets enriched in GOF samples were targets of *Suz12, EED*, and exhibited histone modification of H3K4Me2/H3K27Me3. They are all related to the function of the Polycomb Repressive Complex 2 (PRC2), a well-characterized epigenetic mechanism of gene repression (**Supplementary Figures S3B–F**). A recent study found functional links between lncRNAs, Wnt/β-catenin signaling, and components of PRC2 in liver cancer stem cells (Zhu et al., 2016). The ontology analysis suggests that epigenetic mechanisms may serve as intermediate modulators of Wnt/β-catenin signaling.

Finally, we compared the list of differentially expressed genes in our GOF mouse dermal fibroblast dataset to genes differentially expressed in human biopsied fibrotic tissue such as systemic sclerosis (SSc), desmoid tumors, idiopathic lung fibrosis, and tumor stroma (Hamburg-Shields et al., 2015). Of the genes differentially expressed in GOF mouse dermal fibroblasts compared to controls, we found varying percentages to also be dysregulated in human fibrotic conditions (**Figure 2C**). Our analysis shows that the expression changes in GOF dermal fibroblasts *in vitro* after 48 h of stabilization of β-catenin is consistent with findings from profiling *in vivo* fibrotic mouse dermis in that Wnt/β-catenin signaling may regulate the expression of matrisome genes and contribute to fibrotic conditions (Hamburg-Shields et al., 2015). Therefore, we demonstrate that the Wnt/β-catenin signaling-responsive genes in dermal fibroblasts are also dysregulated in at least one type of human fibrotic tissue.

### LncRNAs *Wincr1* and *Wincr2* Positively Respond to β-catenin Activity in a Tissue-Specific Manner

**FIGURE 3:**
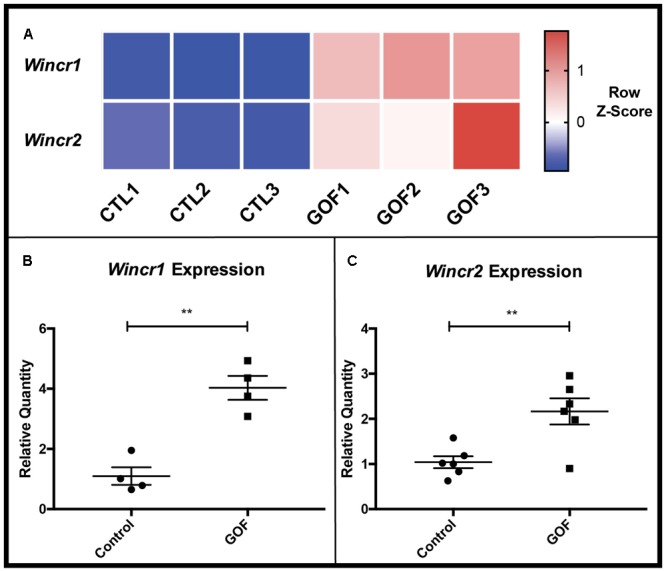
β-catenin stabilized GOF have increased expression of *Wincr1* and *Wincr2*. **(A)** Heatmap representative of RNA-Seq FPKM values of two candidate lncRNAs, *Wincr1* and *Wincr2* from control and GOF dermal fibroblasts (*n* = 3). **(B,C)** Differential expression of lncRNA targets analyzed by qRT-PCR. LncRNA expression levels are significantly up-regulated within *in vitro* β-catenin stabilized GOF fibroblasts vs. the control (*Wincr1*: *n* = 4, *Wincr2: n* = 6). ^∗∗^*P*-value ≤ 0.01.

We next focused on the 111 differentially expressed lncRNAs in the GOF samples as compared to controls. Two top novel candidate Wnt Induced Non-Coding RNAs (*Wincr1* and *Wincr2*) were highly differentially expressed (**Figure 3A**). *Wincr1* isoform 1(ENSMUST00000146678.1) (*GM12603*) was expressed at 37 FPKM in control samples and at 551 FPKM in the GOF samples (average 14x fold-change). *Wincr2 (GM12606)* was expressed at 3.24 FPKM in control samples and 28.27 FPKM in GOF samples, marking over an 8x fold change between conditions. Therefore, we validated increase of *Wincr1* with isoform 1 specific PCR primers and *Wincr2* expression by qRT-PCR in four independent biological replicates of both GOF and controls in P4 ventral dermal fibroblasts *in vitro* (**Figures 3B,C**).

**FIGURE 4:**
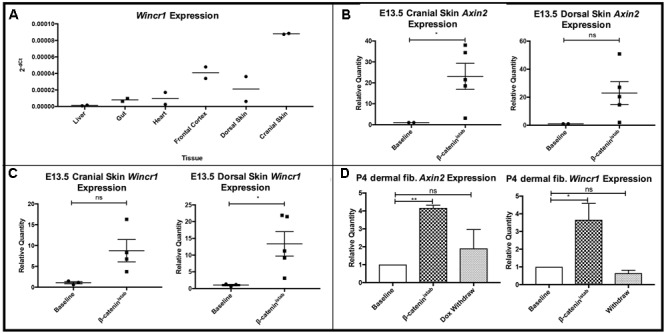
*Wincr1* is differentially expressed in embryonic and perinatal mesenchyme and dynamically responds to changes in β-catenin activity levels. **(A)** Survey of *Wincr1* mRNA levels in embryonic (E13.5) tissues reveals highest expression in embryonic cranial and dorsal skin (*n* = 2). **(B)** *Axin2* expression levels were used to validate β-catenin activity following doxycycline induction for 4 days in *Engrailed1Cre/*+*; Rosa26rtTA/*+*; Teto-*Δ*N89* (β*-cat*^istab^) E13.5 primary fibroblasts *in vitro*. (*P*-value = 0.0237, *n* = 2,5). **(C)** Relative quantity of *Axin2* and *Wincr1* mRNA in β*-cat*^istab^ E13.5 embryonic fibroblasts. **(D)** Relative quantity of *Axin2* and *Wincr1* mRNA in β*-cat*^istab^ P4 fibroblasts following doxycycline induction for 4 days and subsequent withdrawal for 2 days *in vitro*. ^∗^*P*-value ≤ 0.05, ^∗∗^*P*-value ≤ 0.01.

To investigate the tissue-specific expression profile of *Wincr1 in vivo*, we used embryonic skin in which we have previously shown a strong instructive role for Wnt/β-catenin signaling in dermal fibroblast identity and hair follicle initiation (Atit et al., 2006; Ohtola et al., 2008; Fu et al., 2009; Tran et al., 2010; Chen et al., 2012). We assayed for *Wincr1 in vivo* in embryonic cranial and dorsal dermis during hair follicle initiation at E13.5 by qRT-PCR. *Wincr1* displayed a clear tissue-specific expression pattern during normal development in E13.5 mouse embryos. We found *Wincr1* transcript to be abundant in the cranial (head) and dorsal skin, as compared to expression in the embryonic liver, heart, and gut (*n* = 2) (**Figure 4A**). *Wincr1*’s enhanced presence in embryonic cranial and dorsal skin vs. other tissues suggests that our lncRNA candidate could play a role in fibroblast biology. *Wincr2* followed a similar tissue-specific trend in embryonic tissues (**Supplementary Figure S4A**).

### *Wincr1* Dynamically Responds to Wnt/β-catenin Signaling Activity

We further tested β-catenin-responsive expression of *Wincr1* with doxycycline-inducible stabilized β-catenin (β*-cat*^istab^) in E13.5 *Engrailed1Cre/*+*; Rosa26rtTA/*+; *Teto-deltaN89*β*-catenin/*+ cranial and dorsal dermal fibroblasts *in vitro* (Mukherjee et al., 2010). Relative *Axin2* mRNA expression level was measured by qRT-PCR to confirm GOF status after doxycycline administration (**Figure 4B**). In response to β*-cat*^istab^, *Wincr1* was significantly higher in embryonic dorsal and cranial dermal fibroblasts (**Figure 4C**). In the same *in vitro* GOF samples assayed for *Wincr1* expression, an increase in *Wincr2* expression was observed but did not reach statistical significance (**Supplementary Figures S4B,C**).

We tested for dynamic β-catenin-responsive expression of *Wincr1* in doxycycline inducible-reversible levels of β*-cat*^istab^ *in vitro* in P4 ventral dermal fibroblasts. Addition of doxycycline and subsequent withdrawal in culture media allowed us to induce and reverse expression of stabilized β-catenin as shown by *Axin2* mRNA levels in *Engrailed1Cre/*+; *Rosa26rtTA/*+; *Teto-deltaN89*β*-catenin/*+ P4 dermal fibroblasts (**Figure 4D**). Similarly to the previous result, *Wincr1* responded dynamically to the induction and reversal of β*-cat*^istab^ levels (**Figure 4D**), further establishing *Wincr1* as a Wnt/β-catenin signaling-responsive lncRNA in embryonic and perinatal dermal fibroblasts obtained from cranial and trunk skin.

### *Wincr1* Influences Expression of Some Matrisome Genes

We next utilized co-expression network construction to identify and separate putative regulatory targets of both β-catenin and *Wincr1*. It has been shown that correlation of expression between two genes across samples can imply a common regulatory factor (Allocco et al., 2004). Using FPKM values from control and GOF samples, we constructed a network of up-regulated mouse matrisome genes connected on the basis of correlation coefficient (CC > 0.6). Genes were grouped into two clusters: those that correlate with *Axin2* (97 genes), and those that do not correlate with *Axin2* (6 genes). By excluding genes whose expression correlated with that of *Axin2*, a known direct target of β-catenin, we identified a small number of matrisome genes that might be indirect targets of β-catenin regulation. Relevant fibrosis-related marker genes were selected as possessing some or all of the following traits:

(a) Up-regulated in β*-cat*^istab^ dermal fibroblasts by comparison of FPKM (**Figure 1E**)
(b) Inclusion in the Matrisome gene set (**Figure 2B**)
(c) Overexpressed in human fibrotic tissue microarray (**Figure 2C**)
(d) Exclusion from the *Axin2* cluster of the correlation network (**Supplementary Figure S5**)

Of the genes that passed all four criteria, *Matrix Metalloproteinase-10* (*Mmp10*) was selected for functional investigation. Furthermore, there was no significant enrichment of predicted *Tcf/Lef* binding motifs within 5 kb of the transcriptional start site of *Mmp10* and other differentially expressed mRNAs (**Supplementary Figure S2**).

**FIGURE 5:**
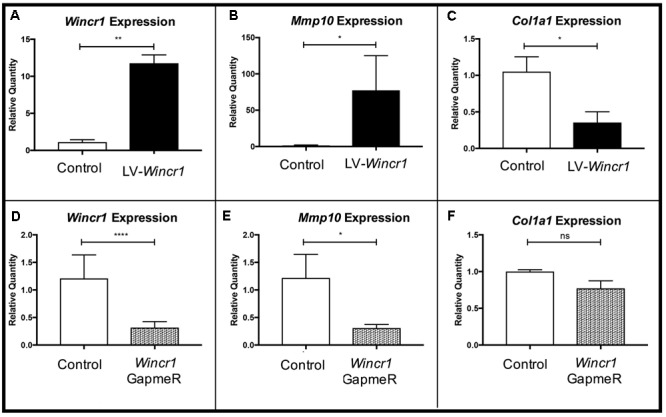
Gene regulatory function of *Wincr1*. **(A,D)** Relative quantity of *Wincr1* RNA is significantly altered as a result of lentivirus or GapmeR infection. **(B,E)** Relative quantity of *Mmp10* mRNA levels correlate with changes in *Wincr1* expression levels. **(C,F)** In LV*-Wincr1*, relative quantity of *Col1a1* is significantly reduced and was comparable between control and *Wincr1* GapmeR (*n* = 5 biological replicates). ^∗^*P*-value ≤ 0.05, ^∗∗^*P*-value ≤ 0.01, ^∗∗∗∗^*P*-value ≤ 0.0001.

To elucidate *Wincr1* function in gene regulation and dermal fibroblast biology, we either overexpressed or knocked down *Wincr1* in P4 ventral dermal fibroblasts *in vitro*. For overexpression, we generated a lentivirus construct to overexpress *Wincr1* (LV-*Wincr1*). For knockdowns of *Wincr1*, we utilized LNA GapmeRs (*Wincr1* GapmeRs). We confirmed increase in *Wincr1* RNA expression levels after infection of LV-*Wincr1* (**Figure 5A**) and reduction after transient transfection with *Wincr1* GapmeR (**Figure 5D**). Dermal fibroblasts with either over- or knocked down expression of *Wincr1* and the parent control cells were then used to investigate changes in the expression of dermal identity and fibrosis-related gene candidates.

We found *Mmp10* mRNA level was highly correlated with levels of *Wincr1* in dermal fibroblasts (**Figures 5A,B,E**). After infection with LV-*Wincr1*, expression of *Mmp10* increased dramatically compared to the control (**Figure 5B**). Conversely, knockdown of *Wincr1* via GapmeRs resulted in a decrease in *Mmp10* expression by ∼30% of basal levels (**Figure 5E**). Also, the expression of *Col1a1*, a key fibrotic marker, correlated negatively with overexpression of *Wincr1* (**Figure 5C**). However, we did not observe a significant difference in *Col1a1* mRNA levels upon knockdown of *Wincr1* (**Figure 5F**). The mRNA levels of markers for fibroblasts identity *Platelet derived growth factor receptor alpha* (*Pdgfra*) and β-catenin responsive genes and fibrotic markers such as *Loxl4, Col5a1*, which were up-regulated in GOF, did not correlate with *Wincr1* mRNA level, indicating that *Wincr1* does not regulate their expression in our system (**Supplementary Figure S6**). Co-expression analysis also identified *Has2* and *Methylthioadenosine phosphorylase* (*Mtap)*, the latter of which shares a locus with *Wincr1*. However, the relative mRNA levels of *Has2* and *Mtap* did not correlate with changes in *Wincr1* level (**Supplementary Figure S4**). Thus, our findings demonstrate that *Mmp10* is a key target of *Wincr1*.

To test if *Wincr1* and β-catenin have synergistic effects on gene expression, we infected *Engrailed1Cre/*+; *R26rTA/*+; *Teto-*β*-catenin* (β*-cat*^istab^) P4 ventral dermal fibroblast with LV*-Wincr1*. We validated induction of *Axin2* in β*-cat*^istab^ condition and confirmed comparable overexpression of *Wincr1* in lentivirus infected β*-cat*^istab^ condition (**Supplementary Figure S7**). *Mmp10* and *Col1a1* mRNA levels in LV*-Wincr1*+ β*-cat*^istab^ were not significantly altered from LV*-Wincr1* only (**Supplementary Figure S5**). These results suggest that Wnt/β-catenin signaling and *Wincr1* do not synergize to regulate *Mmp10* and *Col1a1* mRNA and likely function in a linear pathway.

### *Wincr1* Mitigates Migration and Enhances Collagen Gel Contraction of Dermal Fibroblasts

**FIGURE 6:**
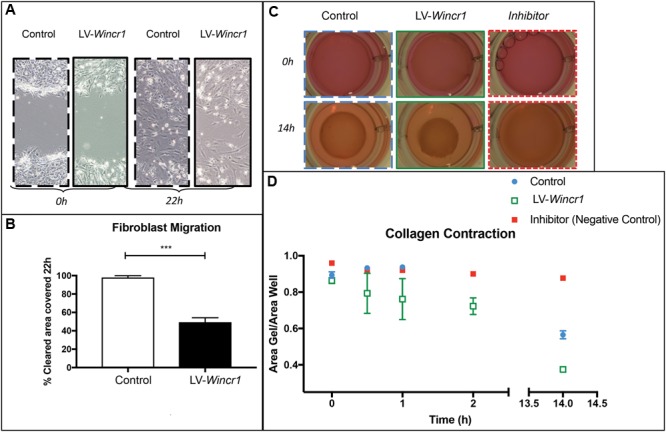
*Wincr1* function in collective cell migration and fibrotic response. **(A)** Dermal fibroblasts with LV*-Wincr1* exhibit decreased migration compared to uninfected controls. **(B)** Migration is significantly diminished after 22 h following a scratch in serum-free media (*n* = 3) and representative of three separate experiments. **(C,D)** Type I collagen gel contraction assay demonstrates LV*-Wincr1* fibroblasts have a higher ability to process collagen. The difference in collagen shrinkage is visible and consistent across three technical replicates from two independent cell lines by 14 h. Points represent the quantitation of collagen gel area normalized to plate area after 14 h using ImageJ software (*n* = 2 between Control and LV-*Wincr1* at 14 h). ^∗∗∗^*P*-value ≤ 0.001.

Given the gene regulatory effect of *Wincr1* on *Mmp10* expression, we sought to determine functional effects of *Wincr1* on cellular behavior of dermal fibroblasts. In order to prevent discrepancies, we used the same stably infected LV-*Wincr1* lines in all functional assays (characterized in **Figure 5A**). Two different dermal fibroblast lines with over-expression of *Wincr1* had comparable rates of proliferation to controls over 7 days (**Supplementary Figure S8**). Next, we used an *in vitro* scratch assay to study the rate of collective cell migration of dermal fibroblasts in a wound healing setting. This multistep process is relevant in many biological processes such as embryonic development and wound healing (Liang et al., 2007). Compared to control, *LV-Wincr1* had attenuated collective cell migration into the cleared area in both serum containing (**Supplementary Figure S8**) and serum-free conditions within 14–22 h (**Figures 6A,B**).

Collagen type1 gel embedded with fibroblasts is considered an *in vitro* model for fibrotic process and matrix turnover (Fang et al., 2004). We determined whether *Wincr1* affects the ability of dermal fibroblasts for collagen contraction, a key fibroblast function. We embedded control and LV*-Wincr1* cells in type I rat tail collagen for 48 h to increase the mechanical load and then released the gels. We found LV*-Wincr1* dermal fibroblasts had much higher capacity for processing collagen than controls (**Figures 6C,D**). The difference in collagen gel shrinkage by 14 h was significant and consistent across the two LV-*Wincr1* cell lines. Thus, gain of function *Wincr1* positively correlated with collagen gel contraction, thereby linking *Wincr1* to a key fibroblast functionality.

In summary, we have identified key mRNAs and lncRNAs that are responsive to Wnt/β-catenin signaling levels and we have demonstrated that a lncRNA responsive to Wnt/β-catenin activity, *Wincr1*, is a potentially new regulator of dermal fibroblast behavior and ECM-related gene expression.

## Discussion

Long non-coding RNAs have emerged as key regulators of many cellular processes, and their dysregulation has been observed in many human diseases (Wan and Wang, 2014). Studies of novel lncRNAs have led to the discovery of new mechanisms of gene regulation paving the way toward novel therapeutic strategies. In our current study, through the genetic stabilization of β-catenin in primary dermal fibroblasts, we identified downstream lncRNAs and mRNAs responsive to Wnt/β-catenin signaling. Our genetically targeted culture systems enabled us to observe the effects of β-catenin’s activity levels on gene expression in dermal fibroblasts without the paracrine influences of other cell types that would be present in whole skin expression analysis. Utilizing this system, we have identified Wnt/β-catenin signaling-induced lncRNAs such as *Wincr1*, that act as putative regulators of key genes such as *Mmp10* and *Col1a1* in ECM biology. *Wincr1* also affects complex cellular behaviors such as collective cell migration and collagen processing and contraction.

Our expression profiling study allowed us to identify genes that were under the influence of Wnt/β-catenin signaling in dermal fibroblasts. Consistent with our previous gene expression analysis from mouse fibrotic dermis after 21 days of sustained Wnt/β-catenin signaling (Hamburg-Shields et al., 2015), gene ontology analysis through functional clustering shows a strong enrichment of ECM and ECM-regulatory gene expression within 72 h of Wnt/β-catenin signaling in dermal fibroblasts. Our analysis also implies that downstream targets of Wnt/β-catenin signaling may alter matrix deposition, remodeling, and fibroblast behavior. Comparison of the Wnt/β-catenin signaling activation gene signature in dermal fibroblasts with those dysregulated in various contexts of human fibrotic diseases confirms that the gene expression signature of β-catenin stabilization in our model is indeed relevant to human fibrotic disease. Thus, β-catenin stabilization in dermal fibroblasts is a relevant signaling pathway in matrix construction and remodeling and a relevant node for therapeutic intervention.

Analysis of the gene expression signature induced by β-catenin stabilization reveals enrichment for PRC2 targets, signifying epigenetic regulation. Unbiased gene set enrichment analysis of genes expressed in dermal fibroblasts shows an enrichment of PRC2 targets. This epigenetic regulatory repressive complex has been shown to interact with lncRNAs, suggesting that a portion of the β-catenin-dependent expression signature controlling ECM and fibrosis-related genes may be regulated by PRC2 or other mechanisms (Rinn and Chang, 2012; Wan and Wang, 2014). While further studies are needed to elucidate the mechanism of β-catenin’s influence on fibrotic genes, these analyses of the coding gene changes following β-catenin activation guided our identification of novel lncRNAs as regulators of gene expression and fibroblast behavior.

Using sustained and inducible-reversible systems of Wnt/β-catenin signaling activation, *Wincr1* was identified as a Wnt/β-catenin signaling induced lncRNA candidate due to its consistent response to β-catenin activity levels in multiple contexts in mouse dermal fibroblasts. Further context-specific functional roles of *Wincr1* will be elucidated in future *in vivo* studies with fibroblast-restricted mutants. Our *in vitro* modulations of *Wincr1* expression levels in dermal fibroblasts demonstrated that this lncRNA has a robust gene regulatory effect on distinct genes. Identification of other *Wincr1* targets will require extensive expression profiling in dermal fibroblasts and other cell types. In addition, our studies reveal that β-catenin stabilization and overexpression of *Wincr1* do not synergize to increase *Mmp10* levels, suggesting that β-catenin and *Wincr1* may function linearly in a uncharacterized genetic or epigenetic pathway to modulate key genes in matrix production and turnover. Our current data show that while *Wincr1* levels are increased after stabilizing β-catenin, *Wincr1* can also independently regulate expression of protein coding genes. Identifying other *Wincr1* targets will allow us to further refine our understanding of how *Wincr1* intersects with Wnt/β-catenin signaling for gene regulation and fibroblast cell behavior. In our current study, we elaborate on the role of *Wincr1* in fibroblast gene regulation and cellular behavior, given that this is a context in which Wnt/β-catenin signaling is known to be important.

Our query of several fibrotic genes suggests that *Wincr1* has a restricted gene regulatory role. Specifically, *Mmp10* expression was consistently responsive to *Wincr1* levels, suggesting it is a putative regulatory target of this novel lncRNA. *Wincr1’s* gene regulatory role does not appear to be pan-ECM, but instead can regulate a defined set of genes. Further mechanistic-based experiments are required to gain insight into how *Wincr1* regulates *Mmp10*, and such studies would be helpful in predicting other targets through binding motifs or other means. *Mmp10/Stromelysin-2* has emerged as a key player in fibrosis and “degradomics” (Overall and Kleifeld, 2006; Schlage et al., 2015; Sokai et al., 2015). MMPs can promote collagen contraction by increasing turnover (Daniels et al., 2003; Bildt et al., 2009), and we found increased contractile behavior in LV*-Wincr1* dermal fibroblasts. *Mmp10* is not a collagenase, but it has the ability to regulate expression of other MMPs, such *as Mmp13*, that have collagenolytic function and promote the resolution of matrix in wound healing. It is not clear if Mmp10 has a similar role in mitigating ECM accumulation in chronic fibrosis settings that are the result of excessive ECM production and inadequate resolution (McKleroy et al., 2013; Rohani et al., 2015). It is tempting to speculate that *Wincr1* participates as a negative feedback to the pro-fibrotic Wnt/β-catenin pathway in dermal fibroblasts. Future functional studies with *Wincr1* in fibrosis models will be needed to demonstrate if it has a fibroprotective function.

Wnt/β-catenin signaling has diverse roles in skin development, tissue homeostasis, and disease. Thus, identifying new genetic and epigenetic regulatory mechanisms will provide novel insight in our understanding of this important pathway in dermal fibroblasts. Discovery of this lncRNA and its preliminary regulatory links to β-catenin signaling, skin development, and known fibrotic protein-coding gene expression indicate that lncRNAs may play an important regulatory role in directing Wnt/β-catenin signaling. This is of particular interest in the context of fibrosis, a condition that can be caused by activation of this pathway, but with gene regulatory intermediates that will require further characterization in order to be of clinical value. The robust responsiveness of *Wincr1* to Wnt/β-catenin signaling indicates the possibility of this and other lncRNAs as targets for therapeutic intervention to treat fibrosis, but also as circulating diagnostic biomarkers (Tang et al., 2015; Vencken et al., 2015; Zhou et al., 2015). Further studies are needed to elucidate the gene regulatory mechanism and *in vivo* functions of *Wincr1* and other β-catenin responsive lncRNAs. Also, mechanistic understanding into *Wincr1* may lead to novel insights into the Wnt signaling pathway and how it regulates key genetic networks throughout embryonic development and adult diseases.

## Author Contributions

NKM, NVM, RA, and AK contributed to experimental design, experiment collection, analysis, writing, and figure preparation. EH-S contributed to experimental design and writing of the manuscript. BI contributed to analysis.

## Conflict of Interest Statement

The authors declare that the research was conducted in the absence of any commercial or financial relationships that could be construed as a potential conflict of interest.

## Footnotes

1 http://primer3plus.com/

2 https://pga.mgh.harvard.edu/primerbank/

3 https://david.ncifcrf.gov/

4 http://opossum.cisreg.ca/oPOSSUM3/

5 http://matrisomeproject.mit.edu/other-resources/

6 http://www.exiqon.com/gapmers

## Supplementary Material

FIGURE S1 Heatmaps of all lncRNAs and mRNAs expressed above 1FPKM. Biological replicates cluster together on the basis of global expression for both **(A)** lncRNA and **(B)** mRNAs by Pearson correlation (based on entities and samples).

FIGURE S2 Enrichment of predicted transcription factor binding site (TFBS) analysis on promoters of mRNA. Enrichment of TFBS within 5 Kb of the transcriptional start site (TSS) of up-regulated **(A)** and differentially expressed (*P*-value = 0.05, fold change > 2) **(B)** mRNAs after activating Wnt/β-catenin signaling. Statistically enriched TFBS (upper right quadrant) are not in the TCF/LEF family of transcription factors associated with Wnt signaling pathway (red color in **A**).

FIGURE S3 Gene set enrichment analysis (GSEA) shows strong enrichment in Polycomb Repressive Complex 2 (PRC2) targets among genes up-regulated after β-catenin stabilization. **(A–E)** Top enriched gene sets from C2 database are Suz12, EED, and PRC2 targets, as well as H3 methylation sites. Gene sets enriched in the β-catenin stabilized GOF condition were plotted based on FWER *P*-value and Normalized Enrichment Score. **(F)** Leading edge analysis of the top four gene sets show common genes driving the enrichment of these signatures in GOF dermal fibroblasts.

FIGURE S4 LncRNA *Wincr2* is differentially expressed in response to β-catenin activity at E13.5. **(A)** Endogenous expression of *Wincr2* within distinct tissues from an E13.5 wild type embryo via qRT-PCR. Steady state mRNA expression level was verified across two independent embryonic litters. **(B)** Relative quantity of *Wincr2* in E13.5 β*-cat*^istab^ cultured cranial and dorsal dermal fibroblasts after 4 days of β-catenin stabilization. **(C)** *Wincr2* expression is not significantly altered after inducing β-catenin stabilization.

FIGURE S5 Co-expression network of genes up-regulated in β-catenin GOF and included in mouse Matrisome. Network includes all genes up-regulated (*P*-value < 0.05, fold change > 2) in GOF samples and included in the mouse Matrisome. Network edges indicate expression correlation between genes, with correlation coefficient above 0.6 shown. Network is separated into clusters based on correlation with *Axin2* expression, or absence of such correlation (to the right).

FIGURE S6 Manipulation of *Wincr1* expression does not affect all pro-fibrotic genes. **(A–G)** Expression of additional matrisome genes as a result of *Wincr1* overexpression. We identified these mRNA targets from the analysis of our dataset, co-expression network generation, known fibroblast identity markers, matrisome gene targets, and literature screens.

FIGURE S7 Lack of synergistic expression of *Mmp10* in cells with LV-*Wincr1* and LV-*Wincr1*+ β*cat*^istab^. **(A)** *Axin2* mRNA levels, a measure of β-catenin activation, is significantly higher in β*cat*^istab^ condition (*P*-value = 0.0152, *n* = 2). **(B)** *Wincr1* level is significantly higher in LV*-Wincr1* and LV*-Wincr1*+ β*cat*^istab^ samples (*n* = 2).**(C)** There is no significant difference in relative quantity of *Mmp10* and *Col1a1* mRNA between LV*-Wincr1* and LV*-Wincr1*+ β*cat*^istab^ samples, (*n* = 2). **(C,D)** Relative quantity of *Col1a1* mRNA is significantly reduced in the presence of LV*-Wincr1* (*P*-value = 0.0023, *n* = 2). Representative of three different experiments. ^∗^*P*-value ≤ 0.05, ^∗∗^*P*-value ≤ 0.01.

FIGURE S8 Overexpression of *Wincr1* does not affect fibroblast proliferation. **(A)** Proliferation curve of control and LV*-Wincr1* infected primary dermal fibroblasts showing comparable rate of proliferation (*n* = 3) and representative of two different experiments. **(B)** Migration is significantly diminished after 15 h following a scratch in the monolayer of LV*-Wincr1* cells in serum-free media (*n* = 3) and representative of two separate experiments.
