# Supplementary figures and images for: Wnt/β-catenin Signaling Pathway Regulates Specific lncRNAs That Impact Dermal Fibroblasts and Skin Fibrosis
Source: Front Genet. 2017 Nov 21;8:183. doi: 10.3389/fgene.2017.00183 (PMC5702388; doi:10.3389/fgene.2017.00183)

A

Color Key  
and Histogram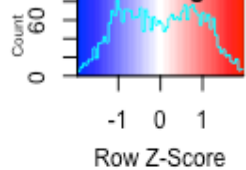

CTL vs. GOF IncRNAs

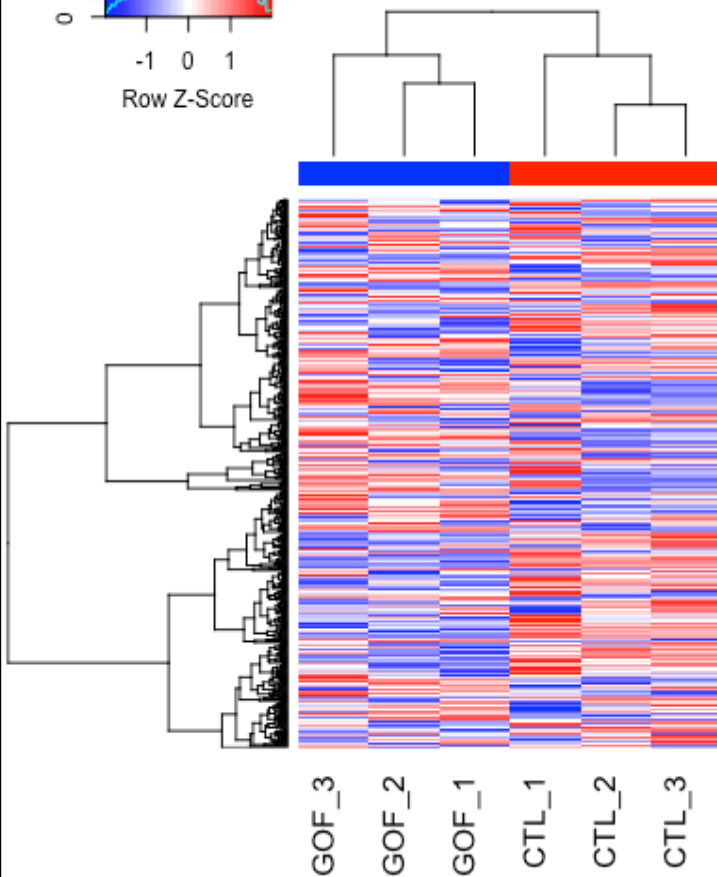

B

Color Key  
and Histogram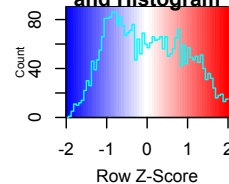

CTL vs. GOF mRNAs

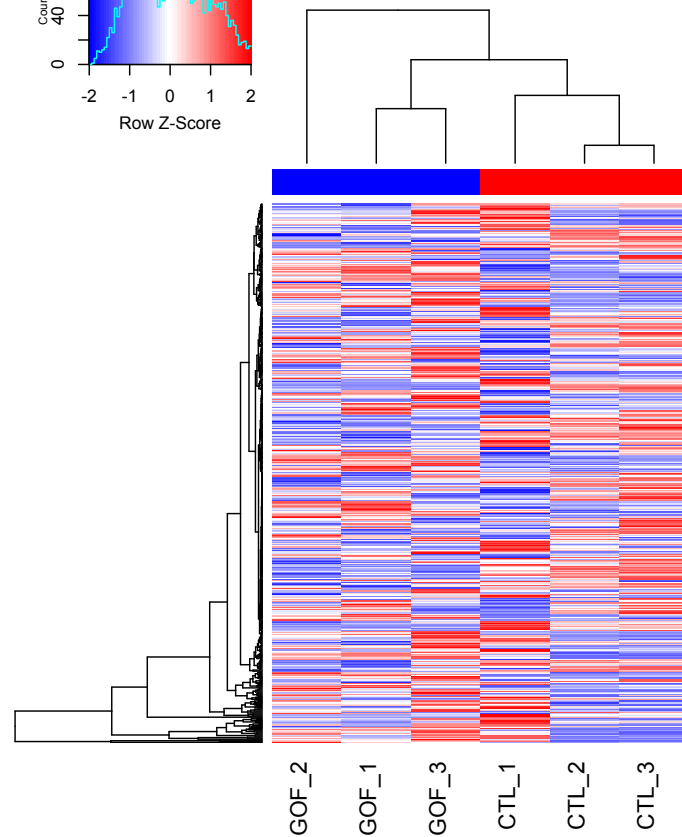

Supplement: FIGURE S1 — Heatmaps of all lncRNAs and mRNAs expressed above 1FPKM. Biological replicates cluster together on the basis of global expression for both (A) lncRNA and (B) mRNAs by Pearson correlation (based on entities and samples). [file Image_1.pdf]

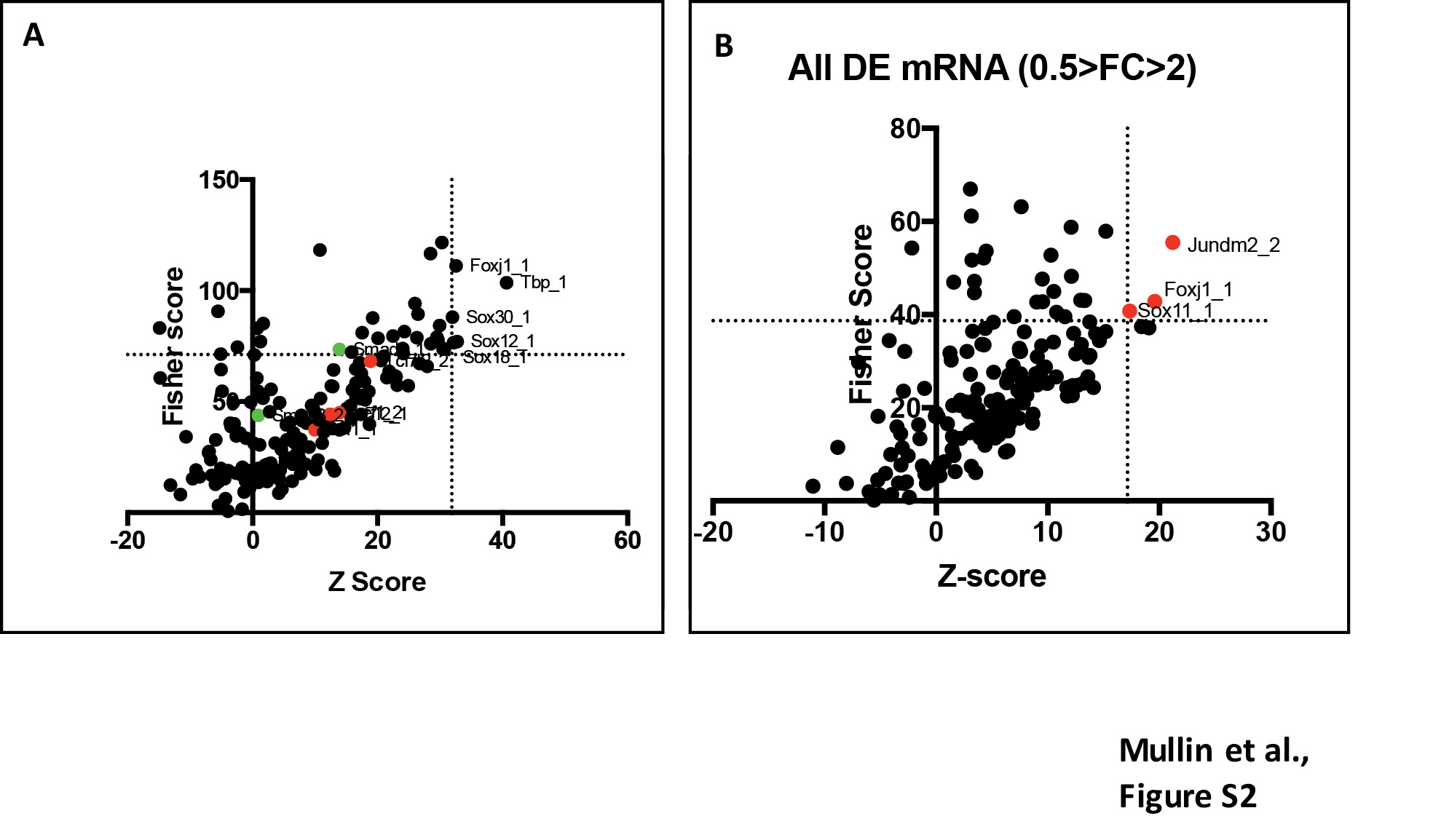

Supplement: FIGURE S2 — Enrichment of predicted transcription factor binding site (TFBS) analysis on promoters of mRNA. Enrichment of TFBS within 5 Kb of the transcriptional start site (TSS) of up-regulated (A) and differentially expressed (P-value = 0.05, fold change > 2) (B) mRNAs after activating Wnt/β-catenin signaling. Statistically enriched TFBS (upper right quadrant) are not in the TCF/LEF family of transcription factors associated with Wnt signaling pathway (red color in A). [file Image_2.JPEG]

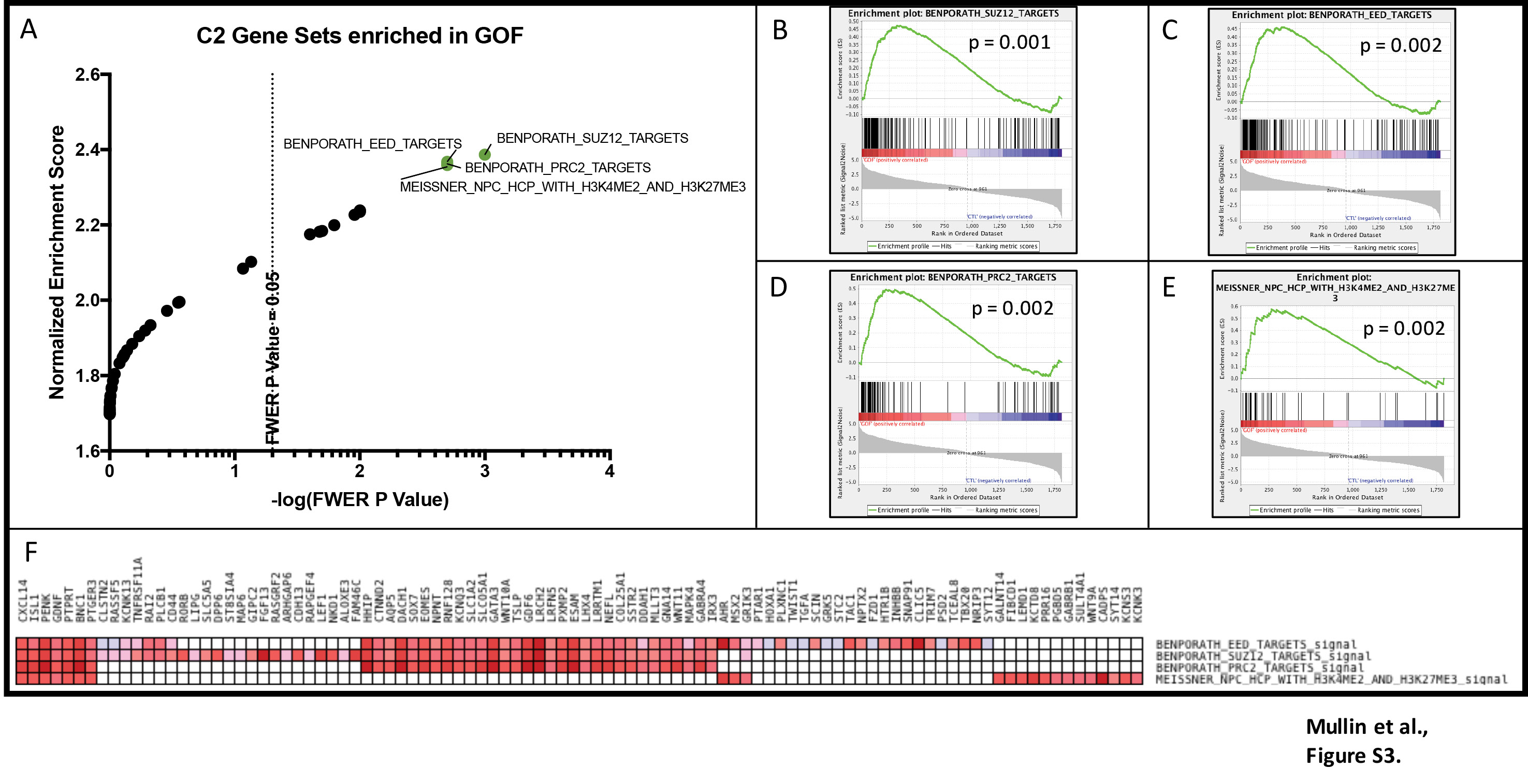

Supplement: FIGURE S3 — Gene set enrichment analysis (GSEA) shows strong enrichment in Polycomb Repressive Complex 2 (PRC2) targets among genes up-regulated after β-catenin stabilization. (A–E) Top enriched gene sets from C2 database are Suz12, EED, and PRC2 targets, as well as H3 methylation sites. Gene sets enriched in the β-catenin stabilized GOF condition were plotted based on FWER P-value and Normalized Enrichment Score. (F) Leading edge analysis of the top four gene sets show common genes driving the enrichment of these signatures in GOF dermal fibroblasts. [file Image_3.JPEG]

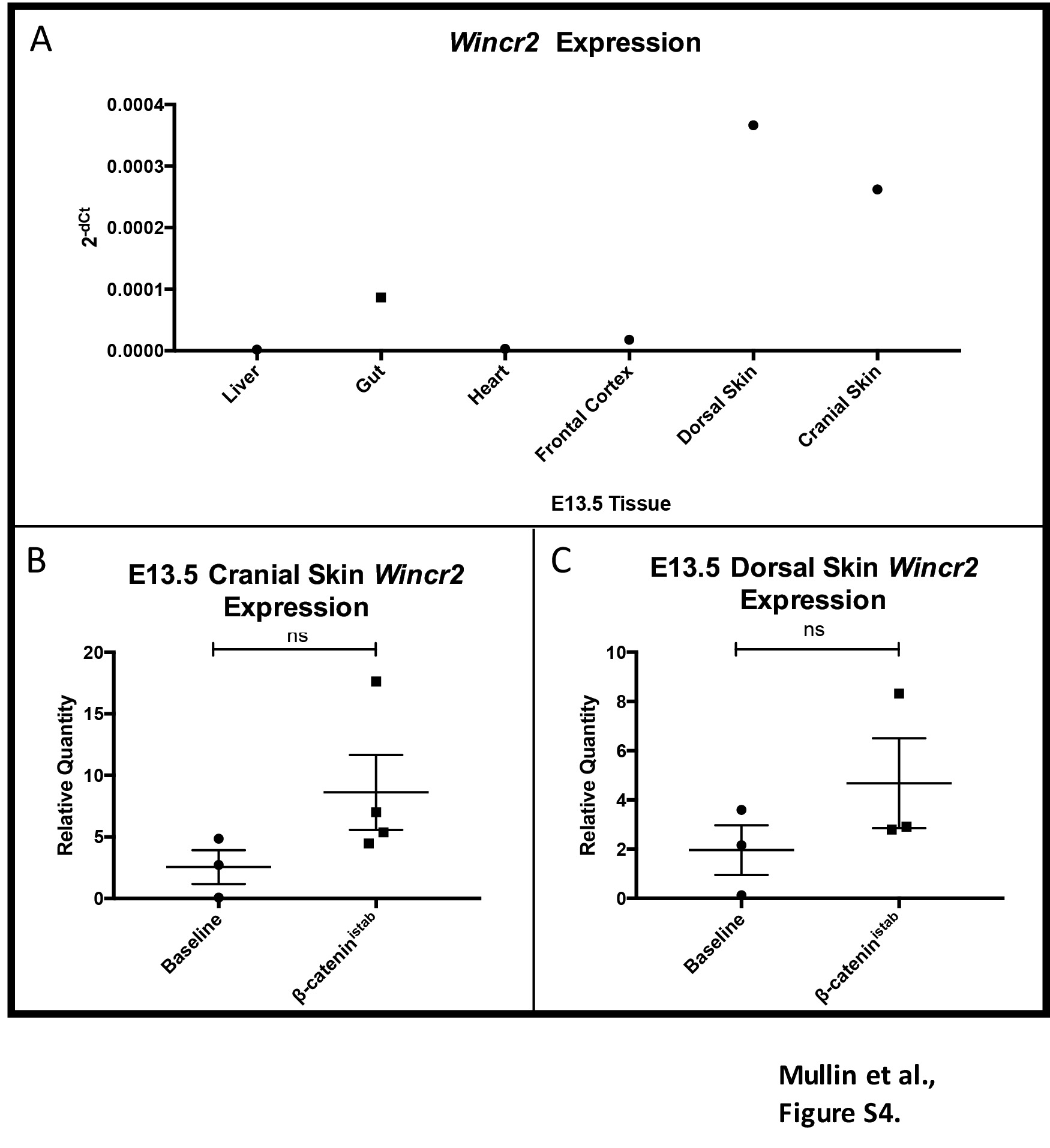

Supplement: FIGURE S4 — LncRNA Wincr2 is differentially expressed in response to β-catenin activity at E13.5. (A) Endogenous expression of Wincr2 within distinct tissues from an E13.5 wild type embryo via qRT-PCR. Steady state mRNA expression level was verified across two independent embryonic litters. (B) Relative quantity of Wincr2 in E13.5 β-catistab cultured cranial and dorsal dermal fibroblasts after 4 days of β-catenin stabilization. (C) Wincr2 expression is not significantly altered after inducing β-catenin stabilization. [file Image_4.JPEG]

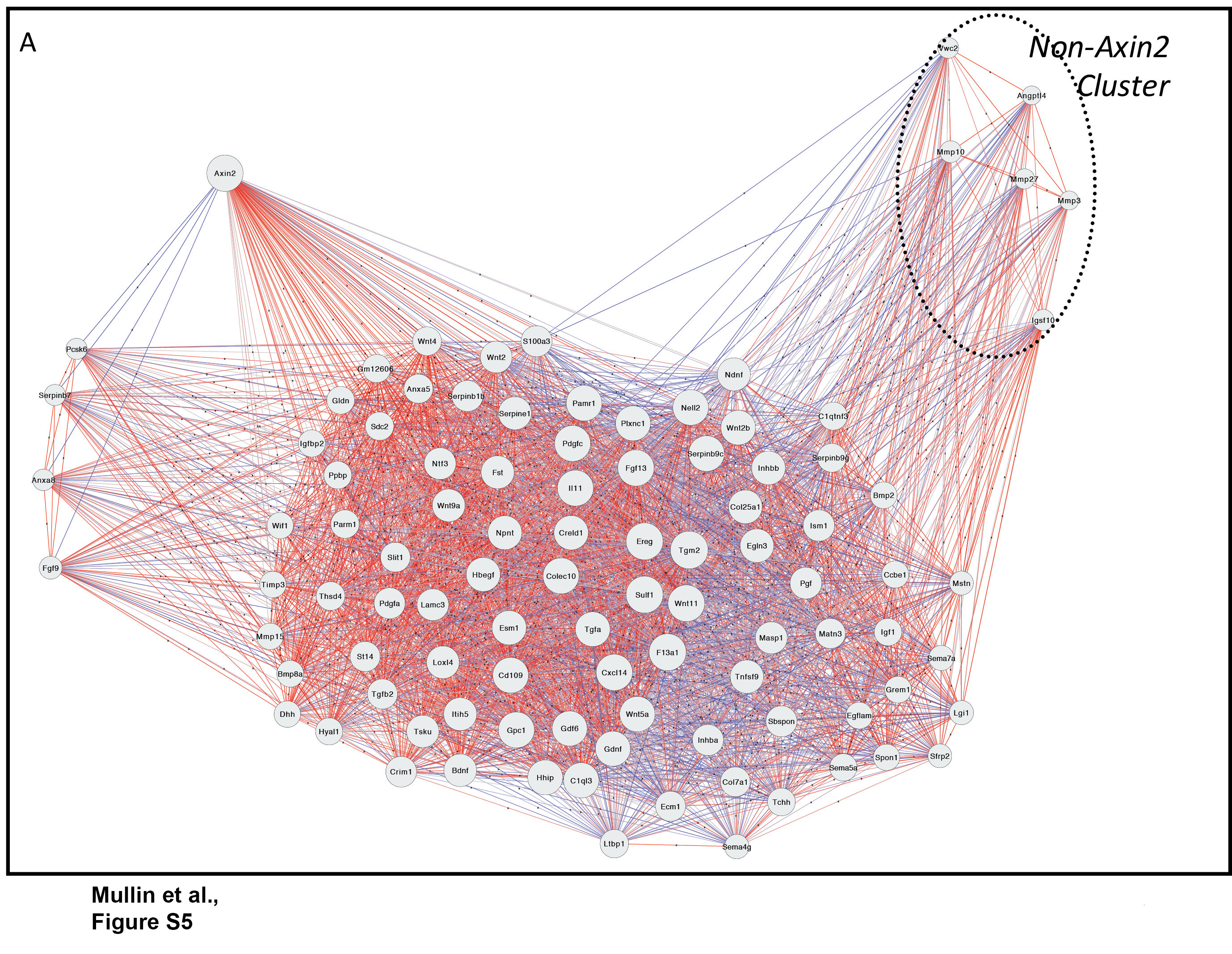

Supplement: FIGURE S5 — Co-expression network of genes up-regulated in β-catenin GOF and included in mouse Matrisome. Network includes all genes up-regulated (P-value < 0.05, fold change > 2) in GOF samples and included in the mouse Matrisome. Network edges indicate expression correlation between genes, with correlation coefficient above 0.6 shown. Network is separated into clusters based on correlation with Axin2 expression, or absence of such correlation (to the right). [file Image_5.JPEG]

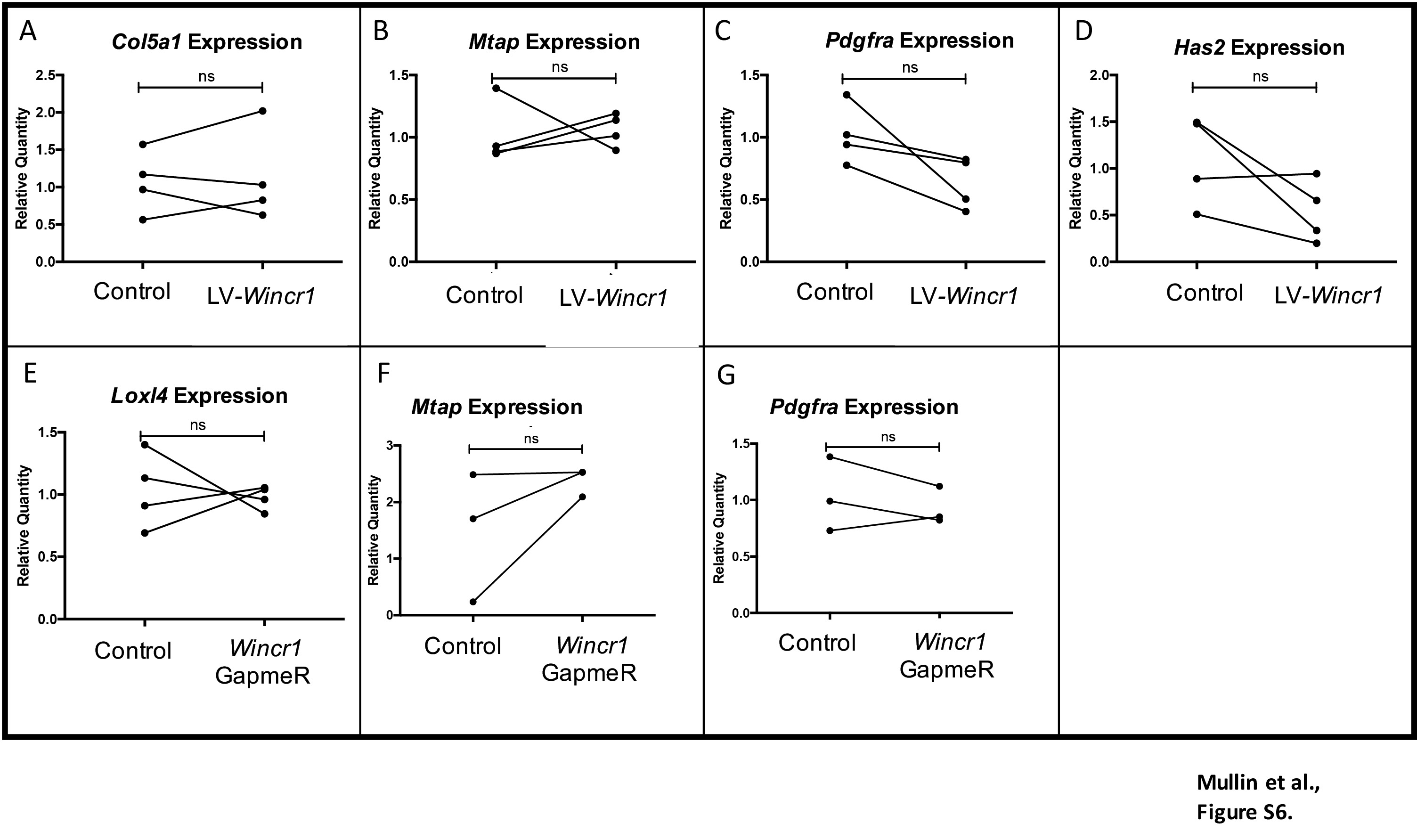

Supplement: FIGURE S6 — Manipulation of Wincr1 expression does not affect all pro-fibrotic genes. (A–G) Expression of additional matrisome genes as a result of Wincr1 overexpression. We identified these mRNA targets from the analysis of our dataset, co-expression network generation, known fibroblast identity markers, matrisome gene targets, and literature screens. [file Image_6.JPEG]

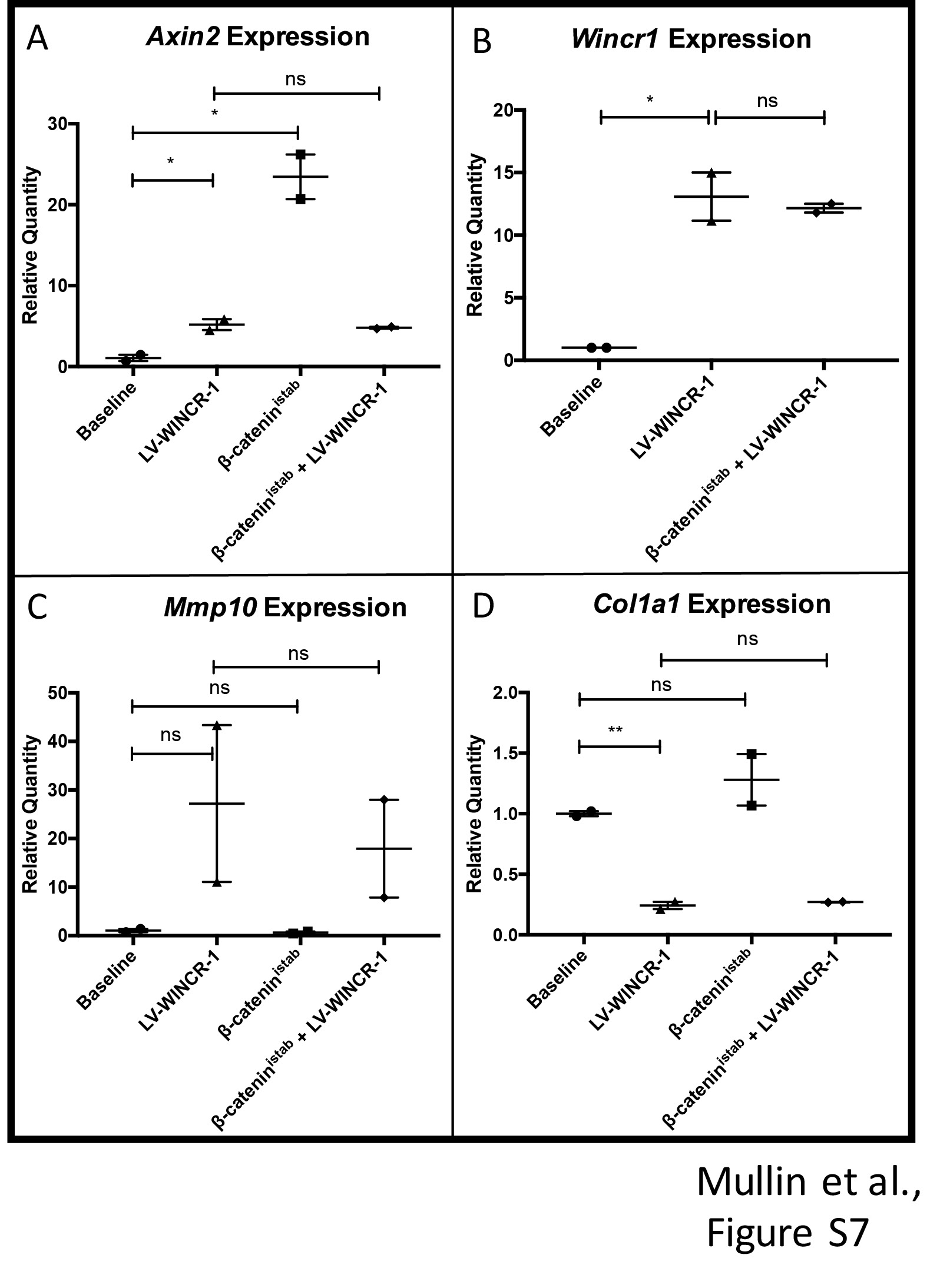

Supplement: FIGURE S7 — Lack of synergistic expression of Mmp10 in cells with LV-Wincr1 and LV-Wincr1+ βcatistab. (A) Axin2 mRNA levels, a measure of β-catenin activation, is significantly higher in βcatistab condition (P-value = 0.0152, n = 2). (B) Wincr1 level is significantly higher in LV-Wincr1 and LV-Wincr1+ βcatistab samples (n = 2).(C) There is no significant difference in relative quantity of Mmp10 and Col1a1 mRNA between LV-Wincr1 and LV-Wincr1+ βcatistab samples, (n = 2). (C,D) Relative quantity of Col1a1 mRNA is significantly reduced in the presence of LV-Wincr1 (P-value = 0.0023, n = 2). Representative of three different experiments. ∗P-value ≤ 0.05, ∗∗P-value ≤ 0.01. [file Image_7.JPEG]

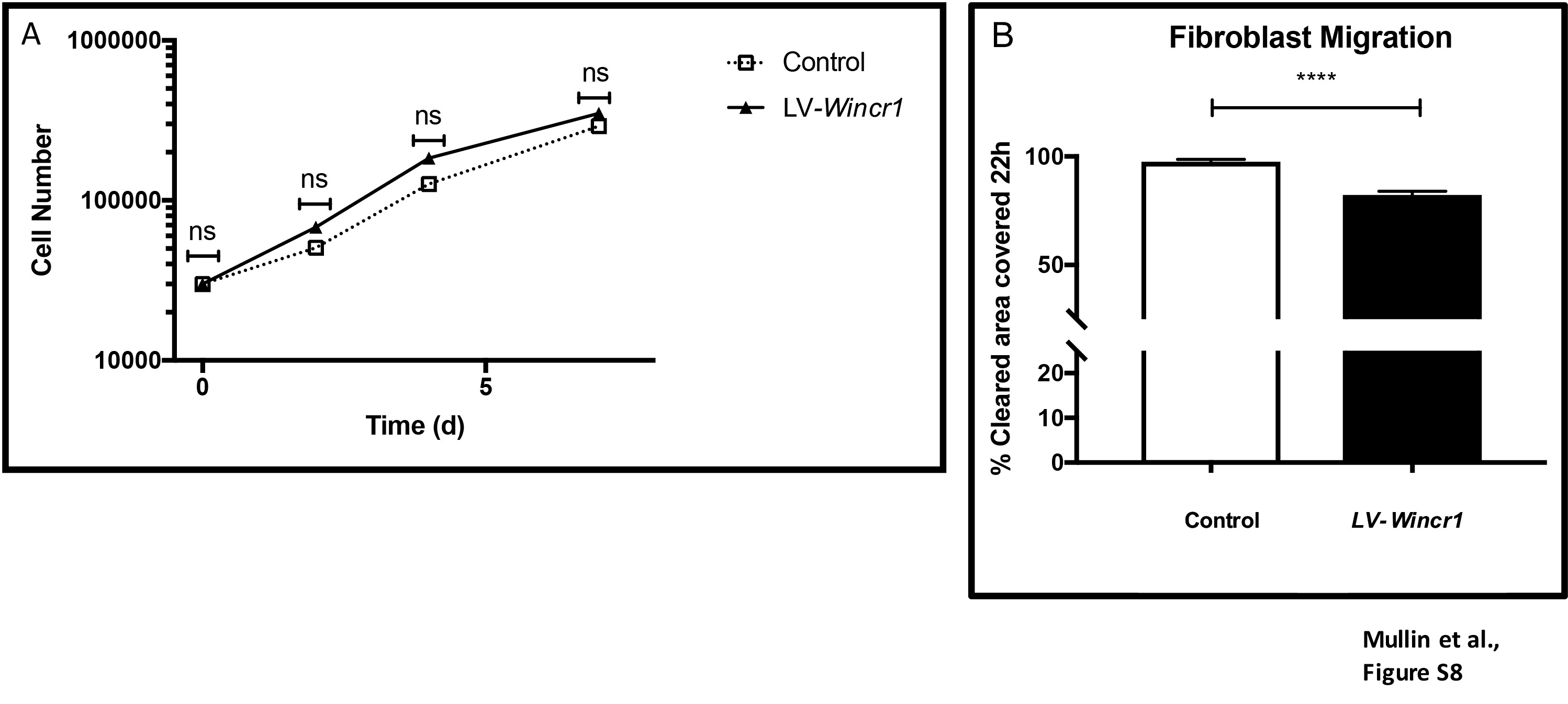

Supplement: FIGURE S8 — Overexpression of Wincr1 does not affect fibroblast proliferation. (A) Proliferation curve of control and LV-Wincr1 infected primary dermal fibroblasts showing comparable rate of proliferation (n = 3) and representative of two different experiments. (B) Migration is significantly diminished after 15 h following a scratch in the monolayer of LV-Wincr1 cells in serum-free media (n = 3) and representative of two separate experiments. [file Image_8.JPEG]
